# Permanent and transient congenital hypothyroidism in Korea: analysis of patients in a single center

**DOI:** 10.1186/1687-9856-2015-S1-P99

**Published:** 2015-04-28

**Authors:** Jeongho Lee, Dong Hwan Lee

**Affiliations:** 1 Department of Pediatrics, Soonchunhyang University Hospital, Seoul, Korea

## Background

Congenital hypothyroidism (CH) is one of the most common preventable causes of mental retardation. Neonatal screening programs allow for the early detection and treatment of CH. Transient CH reverts later to normal, which may or may not require replacement therapy. The aim of this study was to determine the prevalence and manifestations of permanent and transient CH in Korea.

## Methods

We retrospectively reviewed 610 patients who were diagnosed with CH from January 2001 to January 2011 in Soonchunhyang University Hospital. They all underwent clinical re-evaluation after the age of 2-3, based on the thyroid function testing after levothyroxine therapy withdrawl.

## Results

Of the 610 patients diagnosed primarily with CH; 554 (89.1%) patients were diagnosed to have permanent CH, and 66 (10.8%) patients were diagnosed to have transient CH. The median TSH levels before treatment were significantly higher in patients with permanent CH than transient CH (median 58.1:24.1 μIU/mL). Male to female ratio was 1:1.2 (35:31) in transient CH. Of the 66 children diagnosed with transient CH, children discontinued levothyroxine replacement therapy at the age of 25.1±13.2 months. These patients received relatively low dose hormone replacement (initial and at the time of trial of discontinuation).

## Conclusion

We concluded that the incidence of CH as well as the transient form similar to the worldwide reported ones.

